# Steroid Pulse Therapy for Severe Central Nervous System Involvement in Shiga Toxin-Producing *Escherichia coli*-Related Hemolytic Uremic Syndrome

**DOI:** 10.1155/2021/5587050

**Published:** 2021-05-15

**Authors:** Chiara Rosazza, Alberto M Cappellari, Cristiano Gandini, Elisa Scola, Gianluigi Ardissino

**Affiliations:** ^1^Department of Pediatrics, Fondazione IRCCS Ca' Granda Ospedale Maggiore Policlinico, University of Milan, Milan, Italy; ^2^Department of Neuroscience, Fondazione IRCCS Ca' Granda, Ospedale Maggiore Policlinico, University of Milan, Milan, Italy; ^3^Fondazione IRCCS Ca' Granda Ospedale Maggiore Policlinico, Anestesia e Terapia Intensiva Donna-Bambino, Milan, Italy; ^4^Neuroradiology Unit, Fondazione IRCCS Ca' Granda Ospedale Maggiore Policlinico, Milan, Italy; ^5^Center for HUS Prevention,Control and Management, Fondazione IRCCS Ca' Granda Ospedale Maggiore Policlinico, Milan, Italy

## Abstract

We report on the case of a 7-year-old boy with Shiga toxin-producing *Escherichia coli*-related hemolytic uremic syndrome (STEC-HUS), initially presenting with abdominal pain as the only clinical feature and thus requiring differential diagnosis with a surgical emergency. Diagnosis of STEC-HUS was made with the appearance of bloody diarrhea and renal function impairment, and the clinical picture rapidly progressed to multiorgan failure. Relatively late and severe central nervous system (CNS) involvement was present, characterized by subacute encephalitis progressing to coma, which became apparent when the acute phase of thrombotic microangiopathy was resolving. Therefore, neurologic manifestations were thought to be related to reperfusion damage to the CNS and high-dose IV steroid pulse therapy was empirically administered. Following this therapeutic scheme, neurologic involvement resolved with no sequelae. This case offers several points of discussion on the clinical presentation and the diagnostic approach to STEC-HUS, on the related neurologic complications, and on a novel approach to their management.

## 1. Introduction

Shiga toxin-related HUS (STEC-HUS) is a common thrombotic microangiopathy (TMA), caused by enterohemorrhagic *Escherichia coli*, accounting for approximately 85% of HUS cases in children [1]. Organs, other than the kidney, may be severely affected in STEC-HUS including the central nervous system (CNS) [2]. We report a case of STEC-HUS, and we discuss the nonconventional use of pulse steroid treatment for the severe CNS involvement.

## 2. Case Report

**Figure 1:**
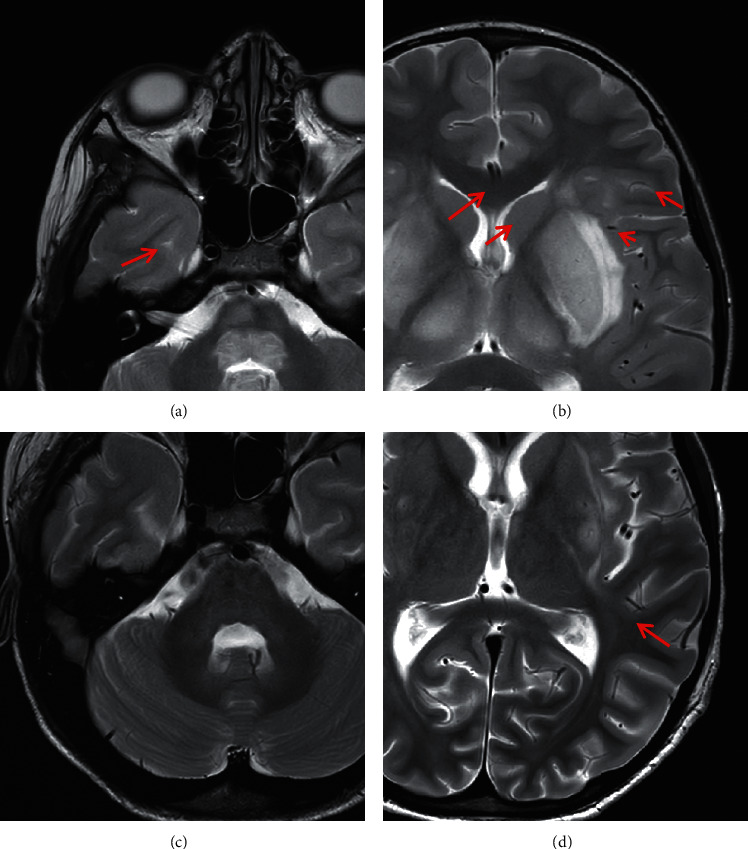
MRI scan at presentation (a, b) and at two weeks follow-up (c, d). In a and b, axial T2 weighted images show hyperintense signal changes in the tegmentum of the pons (a, red arrow), in the thalami (b, red arrows), in the putamen bilaterally with involvement of external capsule, claustrum, and capsula extrema (b, red arrows), more prominent in the left side. In c and d, the follow-up MRI scan shows a regression of the signal changes with the exception of the persistence of a small area of hyperintensity in the left putamen and external capsula (d, red arrow).

A previously healthy 7-year-old boy (R.) was brought to the Pediatric Emergency Dept. for intermittent periumbilical abdominal pain associated with a single episode of vomiting and no diarrhea. On admission his temperature was 38.3°C, rebound tenderness was present in right iliac fossa, and blood tests showed an increase in WBC with neutrophilia and a CRP of 5 mg/dl. The remaining laboratory results were within the normal range (serum creatinine (sCr) 0.56 mg/dl, PLT 227000/mm^3^). The following morning the child opened his bowels once with bloody diarrhea (BD). Because of persistent abdominal pain, explorative laparoscopy was performed on that same day and the appendix was removed. Approximately 24 hours after surgery, R. became anuric; laboratory findings showed hemolysis (Hb from 16.0 to 13.8 g/dl, LDH 3860 U/L), thrombocytopenia (PLT 74000/mm^3^), and increased sCr (1.85 mg/dl). Genes for Shiga-toxin 1, 2 and intimin were identified on a stool sample and *E. coli* O157:H7 was isolated on stool culture, thus confirming the diagnosis of STEC-HUS, and R. was referred to our unit. After admission, R. developed multiorgan failure. The clinical picture rapidly evolved to septic shock requiring inotropes, vasopressors, and orotracheal intubation. Broad spectrum antibiotic therapy with meropenem, vancomycin, and azithromycin was started. On day 10, the patient developed nonfluent dysarthric speech, horizontal gaze palsy, and subacute paraplegia followed by quadriplegia, convulsive seizures, and coma. Brain MRI showed thalamic and subcortical lesions consistent with microvascular ischemic damage and widespread vasogenic edema of midbrain, pons, posterior medulla, thalamus, and basal ganglia (Figure 1).

Electroencephalography (EEG) showed slow background activity, diffuse paroxysmal discharges, and polyspikes associated with myoclonic jerks of the head, which were treated with phenobarbital 15 mg/kg/day, followed by 5 mg/kg/day.

R. was then started on high-dose IV steroid therapy (methylprednisolone 30 mg/kg/day) for 6 days, followed by oral prednisone 2 mg/kg/day, slowly tapered over the following 30 days. Additionally, he was administered Eculizumab (300 mg) because of markedly reduced C3 (60 mg/dL). Genetic testing for atypical HUS was negative. Neurological conditions slowly improved with full recovery within a month. Ocular involvement was also present, with an impairment of visual acuity related to intraretinal hemorrhages (Purtscher-like retinopathy). Serial evaluations of fundus oculi showed gradual reabsorption of hemorrhagic lesions and improvement in macular edema.

Renal failure with anuria required extracorporeal renal replacement therapy. In the meantime, TMA gradually resolved and platelet count normalized by day 10. During the 3^rd^ week of disease, R. developed hypertension, and towards the end of the 2nd month of disease, he showed an acute confusional state with horizontal nystagmus; an emergency CT scan confirmed the diagnosis of posterior reversible encephalopathy syndrome (PRES). IV sodium nitroprusside provided a better BP control and gradual resolution of neurologic manifestations.

One month after surgery, a 2nd explorative laparoscopy was necessary due to pneumoperitoneum caused by incompetence of the appendiceal stump.

R. also experienced acute pancreatitis (amylase 370 U/L and lipase 1239 U/L) associated with abdominal pain (epigastric and periumbilical) and multiple episodes of bilious vomiting. Kept nil per os, on total parenteral nutrition, the child recovered in 2 weeks. Finally, poor glycemic control emerged as an additional problem, and R. was started on insulin, which was stopped following steroid discontinuation. R. was discharged at the end of the 9th week of disease, and he regularly attends our center as an outpatient to continue hemodialysis.

## 3. Discussion

We report on a case of STEC-HUS, initially presenting with abdominal pain as the only clinical feature, thus wrongfully addressed to surgery with severe and life-threatening consequences. The most common reported indications for surgical referral in patients who later developed STEC-HUS are intestinal intussusception, appendicitis, or inflammatory bowel disease, and this often happens in patients with BD before STEC infection has been ruled out by Shiga-toxin determination. In this setting, surgery is likely to worsen the clinical picture because of the increased inflammation, bleeding risks (as soon as platelets count drops), and use of potentially nephrotoxic drugs. In addition, surgery eventually refrains patients from receiving appropriate treatment, such as volume expansion, which was proven to mitigate disease severity [3]. Indeed, the Hb level in our patient at presentation of TMA was as high as 16 gr/dL, clearly indicating hemoconcentration and thus anticipating a more severe course [4]. We underline that whenever a patient has BD, the search for Shiga-toxin in stools is mandatory before other diagnostic options are considered, given that almost 5% of BDs in children are related to STEC infection. Clinically evident extrarenal involvement in STEC-HUS is not rare, given the systemic nature of the TMA, and can virtually involve any organ, but neurological involvement is the most frequent (20–50% of cases) and represents the leading cause of death [2–5]. Its pathogenesis is related to the combination of hypoxia, ischemia, and inflammation, although hypertension, electrolyte imbalances, and metabolic disorders may also play a role [5, 6].

It was recently suggested that Shiga-toxin cannot directly damage neuronal cells, as these do not express the specific Gb3/CD77 receptor. In a rabbit model treated with purified Shiga-toxin 2, neurodegenerative events were shown to be related to the inflammatory response [7]. Furthermore, in an in vitro study on human astrocytes, the stimulation with Shiga-toxin 2 induced the release of proinflammatory chemokines (particularly IL8) possibly responsible for neuronal damage. The authors speculate that steroid therapy should be considered in STEC-HUS with CNS involvement, for its downregulating activity on inflammatory cytokines production [8].

Finally, in late onset STEC-HUS-related neurological manifestations, an immune-mediated pathogenetic mechanism was also advocated [9].

Clinical presentation of cerebral damage in HUS is extremely variable, ranging from mild alteration of consciousness to seizures, hemiparesis, altered pyramidal syndrome, extrapyramidal syndrome with hypertonia and coma, disphasia, diplopia, and facial palsy. It may be useful to classify CNS involvement in STEC-HUS in early (within the initial 5 days of illness) and late onset (thereafter). The early onset is more likely due to ischemia/hypoxia (related to microvascular thrombosis) or hyponatremia (either spontaneous or iatrogenic in a patient infused with hypotonic solution when AKI sets in). Late onset neurologic involvement includes reperfusion injury and PRES, representing a complication rather than a manifestation of disease-specific damage [10].

Brain MRI is the imaging technique of choice for the assessment of patients with CNS involvement during HUS, and a typical pattern of early onset is characterized by abnormal signal in the basal ganglia and thalami. The signal changes may involve the white matter tracts of the internal and external capsule, the brain stem, and the cerebellum as well. The pattern of lesions on MRI does not seem to have a role in predicting neurological outcome [10].

Long-term neurological sequelae in STEC-HUS are described in up to 40% of cases with CNS involvement, with motor functions being predominantly affected [11], and current treatment remains symptomatic, consisting in the support to vital functions and the use of antiepileptic drugs to control seizures [2]. Steroids and Eculizumab have been tried in severe cases, with variable evidence of treatment success. In our case the CNS involvement developed relatively late, when the increase in platelet count was already anticipating the recovery from TMA. Such severe and late neurological symptoms suggested reperfusion injury rather than a primary damage, which should have presented much earlier. High-dose IV steroid treatment was administered and a full neurological recovery was obtained.

Data currently available regarding the efficacy of steroids in treating CNS involvement in STEC-HUS are limited. In a murine model exposed to sublethal doses of Shiga-toxin 2, neurodegeneration in the motor cortex and neuronal damage significantly decreased after treatment with a single dose of dexamethasone [12]. In a randomized controlled trial, oral methylprednisolone (5 mg/kg/day for 7 days) did not improve neurological parameters during the acute phase of HUS in comparison to placebo [13]. When high-dose IV steroid therapy is considered, data mainly derive from rare case reports and they are not univocal. Yada et al. successfully treated a 14-year-old girl with STEC-HUS and acute encephalopathy with high-dose steroid pulse therapy (two courses of IV methylprednisolone 500 mg/day for 3 days) in association with plasma exchange, obtaining full recovery [14]. Hosaka et al. described a 20-year-old woman who developed severe alteration of consciousness leading to coma and was treated with methylprednisolone pulse therapy (1000 mg/day for 3 days), with rapid clinical improvement and complete resolution of deep grey matter lesions on brain MRI [15]. On the contrary, Imataka et al. reported on a 22-month-old boy unsuccessfully (cerebral death) treated with brain hypothermic therapy and steroid pulse therapy [16].

In conclusion, our experience suggests that steroid pulse therapy should be considered as a potentially life-saving therapeutic resource in STEC HUS, particularly in case of delayed CNS involvement, and explored accordingly with well-designed studies.

## Data Availability

No data were used to support this study.
